# Factors Correlated with Success Rate of Outpatient Smoking Cessation Services in Taiwan

**DOI:** 10.3390/ijerph15061218

**Published:** 2018-06-10

**Authors:** Wei-Hsin Huang, Hsin-Yin Hsu, Betty Chia-Chen Chang, Fong-Ching Chang

**Affiliations:** 1 Department of Family Medicine, Mackay Memorial Hospital, Taipei 106, Taiwan; whh.5881@mmh.org.tw (W.-H.H.); camelhsu@gmail.com (H.-Y.H.); betty905@gmail.com (B.C.-C.C.); 2 Department of Health Promotion and Health Education, National Taiwan Normal University, Taipei 106, Taiwan

**Keywords:** smoking cessation, outpatient smoking cessation service, success rate

## Abstract

Smoking is the leading cause of preventable death. The purpose of this study was to explore the patient’s and physician’s factors that are correlated with smoking cessation success rate. A total of 877 smokers who visited the outpatient smoking cessation services at a medical center in Northern Taiwan were recruited for the study. Phone interviews were carried out six months after the initial visit to evaluate the success rate of smoking cessation. The result showed that the abstinence rate at six-month was 37.7%. By the multivariate logistic regression model, the predictive factors of abstinence were smokers who had a lower Fagerström test for cigarette dependence (FTCD), lower exhaled carbon monoxide (CO) concentration, or who smoked less than 20 cigarettes per day at the first visit. Smokers who had more than one smoking cessation outpatient visit or seen by physicians who, on average, delivered more than one smoking cessation consultations per week also led to a higher success rate. Therefore, we suggest that physicians should put more efforts and encourage follow-up visits for some smokers by knowing their characteristics at the first visit. Furthermore, physicians with more experience in smoking cessation consultation seemed to be more likely to help patients to quit smoking successfully.

## 1. Introduction

Lowering smoking rate is still a challenge faced by public health. Tobacco control in Taiwan implemented MPOWER measures recommended by the WHO, and Taiwan attained the highest scores for a total of four items, namely: monitoring of tobacco use and policies, protection from tobacco exposure, offering help to quit tobacco use, and enforcing bans on tobacco advertising, promotion, and sponsorship [1]. New regulations under the Tobacco Hazard Prevention Act had gone into effect on 11 January 2009. It was considered as a major public health accomplishment in Taiwan where smoking rates among adults over the age of 18 fell from 21.9% in 2008 (male 38.6%, female 4.8%) to 17.1% in 2015 (male 29.9%, female 4.2%), and it was estimated that the smoking population had decreased by 760,000 over seven years [1].

Since 2002, the Health Promotion Administration (HPA) of the Ministry of Health and Welfare in Taiwan had provided subsidies for smoking cessation services. Smokers above 18 years of age with scoring at least four points on the Fagerström test for cigarette dependence (FTCD) (previously named Fagerström test for nicotine dependence (FTND) [2]) or smoking 10 or more cigarettes per day may undergo two treatment courses each year. Each treatment course provides up to eight weeks of medication, counseling, and subsidies for each clinic visit. But, with only fixed subsidies for smoking cessation medication (NT$ 250 per week), smokers may still have to pay NT$ 550–1250 (US$ 18.3–41.7) each week, which would be considered as too much for a low-income population. To increase the utility of smoking cessation clinics, the HPA has launched the second-generation smoking cessation policy in 2012, where a maximum co-payment of only NT$ 200 (US$ 6.7) would be required. The total number of people using the services in Taiwan increased over the years, from 48,765 in 2011 to 153,148 in 2015, and the six-month cessation success rate was 26.5% in 2015 [1].

The effectiveness of smoking cessation interventions based on latest Cochrane Review showed physician’s advice, nursing intervention, individual behavioral counseling, group behavior therapy telephone counseling, and provision of medicine all increased the chances of quitting successfully [3]. Studies have shown policies that included an increase of health care provider incentives and medication subsidies have successfully promoted smoking cessation services [4,5], and reimbursement for smoking cessation treatment is efficacious in increasing the use of services [6]. However, few studies examined the impact and factors related to smoking cessation after implementing medication subsidies policies for smoking cessation outpatient services at medical centers. It is important to identify the factors that are correlated to cessation success rate for health care providers to improve the quality of smoking cessation services. Understanding the factors may increase the effectiveness of public health programs that aim to encourage smoking cessation.

## 2. Materials and Methods

### 2.1. Study Population

A total of 877 smokers who used the outpatient smoking cessation services at a medical center in Northern Taiwan between 1 January and 31 December 2014 were recruited for the study. Participants were over the age of 18 years old and were legally covered in the National Health Insurance Program in Taiwan, with FTCD score equal to or greater than four or smoked more than 10 cigarettes per day. All of the participants received pharmacotherapy and counseling for smoking cessation from physicians. Medication, including varenicline and nicotine replace therapy (NRT), was subsidized by the government. Counseling included physician’s advice and provision of brief handouts. Patients were prescribed a range of one to four weeks of medication during each clinic visit, depending on the condition of the patients. Each patient may receive a maximum of sixteen weeks of smoking cessation services in one year. The study was performed with the approval of the institutional review board of Mackay Memorial Hospital, Taipei, Taiwan (application number, 17MMHIS049).

### 2.2. Data Collection and Outcome Measure

The primary outcome was smoking cessation success at six months. We defined smoking cessation success by the seven-day point abstinence, where the participants were asked whether they had smoked at all over the past seven days. To evaluate the success or the failure in smoking cessation, phone interviews by a well-trained nurse were scheduled six months after the first visit of smoking cessation service. Those who reported no smoking were classified as success, and any smoking behavior in the past seven days or loss to follow-up were classified as failure. This definition of success and failure were in accordance with that used by the HPA.

After reviewing articles [4,7,8,9,10], we selected variables and measures that were linked to the primary outcome. At the first visit, each participant completed a questionnaire collecting demographic information, the number of smoking years, the average number of cigarettes smoked per day, nicotine dependence level (measured by the FTCD), and the exhaled air carbon monoxide (CO) concentration (ppm) (measured by a CO device). We also collected data on the number of clinic visits for the current treatment course, the type of medication (varenicline or NRT) used for smoking cessation, and physicians’ factors, including the number of years in clinical practice, gender, and the amount of experience in smoking cessation (measured by the number of smoking cessation consultations physician average provided per week).

### 2.3. Statistical Analyses

SPSS software version 20 was used for all statistical analyses. Continuous variables, such as age and CO values, were expressed as mean (SD). Categorical variables included total smoking years (≥20 years or <20 years), average number of cigarettes per day (≥20 or <20 cigarettes per day), FTCD level (low: 0–3, medium: 4–6, high: 7–10), number of clinic visits (≥two times or one time), use of medication (varenicline or NRT), and the characteristics of the physician. The Chi-square test was used to analyze the difference of 6-month smoking cessation rate among each of these categorical variables. These univariate analyses help us to select variables that predicted abstinence at six months. All variables that predicted abstinence at 6 months in univariate analyses were entered into a multiple logistic regression analysis, controlling the potential confounders, including age and gender. Results were presented as odds ratios (OR) with 95% CI. Statistical significance was set at *p* < 0.05.

## 3. Results

Characteristics of the patients and physicians and their association with six-month smoking cessation success are shown in Table 1. The participants recruited were mainly male (77.8%), with a mean age of 45.3 years. Our results showed that the six-month point abstinence rate was 37.7% (331/877). Older smokers seemed to have a higher success rate (*p* = 0.031). Univariate analyses showed the following patient’s factors to be associated with a higher 6-month cessation success rate: smoking less than 20 cigarettes per day (*p* = 0.001), lower FTCD level (*p* < 0.001), lower exhaled CO value at first visit (*p* < 0.001), and more than one visit for smoking cessation service (*p* = 0.026). There were no significant difference in cessation rates among smokers based on the total smoking years (≥20 years or <20 years) and the use of medication (varenicline or NRT). For the physician’s factors, smokers seen by physicians who average delivered more than one smoking cessation consultations per week were more likely to quit smoking successfully than smokers that were seen by physicians who delivered less than one consultation per week (40.6% vs. 27.6%, *p* = 0.001). However, no correlations were noted for either gender or the duration of physician’s clinical practice with the six-month cessation success rate.

All the variables that were associated with abstinence at six months in the univariate analyses were entered into a multiple logistic regression analysis (Table 2). After adjusting for gender and age, average number of cigarettes smoked each day, FTCD levels, exhaled CO value at first visit, number of clinic visits, and physician’s consultation frequency in smoking cessation service were independent predictors for success rate in smoking cessation. We also found that there existed dose-response relationship in FTCD levels. Physicians who were more experienced in smoking cessation consultation had a higher chance to help their patients to quit smoking successfully.

## 4. Discussion

Providing appropriate smoking cessation counseling and increasing the smoking cessation success rates are of utmost importance for health professionals. A better understanding of the patients’ and physicians’ factors correlated with success rate may increase the effectiveness of counseling. In addition to previously known factors, including the average number of cigarettes smoked each day, FTCD levels, and exhaled CO value at first visit, our study also found that number of clinic visits and physician’s experience in smoking cessation consultation to be independent predictors for success in smoking cessation. Effective counseling is an essential factor for successful smoking cessation. One meta-analysis that examined the appropriateness of cessation counseling showed in-person counseling sessions, both physician and nonphysician counselors, the number of counseling sessions, and the duration of counseling sessions were related to success in smoking cessation six months after the initiation of intervention [11]. The latest Cochrane Review even showed physician’s intensive advice for smoking cessation are more effective than brief advice (OR = 1.37, 95% CI = 1.20–1.56) [3]. The smoking cessation consultations given in our study was a series of brief clinic sessions during an eight-week treatment course. The consultation included physician’s advice and the provision of brief handouts, and physicians would book the next follow-up visit for the patients. One study in Taiwan indicated that an increase of subsidies for smoking cessation services, male gender, older age, daily smoker, having previous attempts at smoking cessation, perceiving oneself as having poor health, and being aware of the benefits of smoking cessation services were significantly positively associated with receiving smoking cessation counseling from health professionals [12]. Age and gender were the two most widely discussed demographic predictors. Many studies showed that older smokers are more likely to quit successfully [4,10,13,14], however, contradicting conclusion also existed [15]. Studies that examined gender differences in smoking cessation had produced mixed results. One review demonstrated that women have more difficulty maintaining long-term abstinence than men [16]. A study on smoking cessation services in Taiwan also showed that women were significantly less likely than men to be abstinent at one year and three years [17]. The poor long-term outcome in women had been generally attributed to greater concerns about weight gain. As a matter of fact, cigarette smoking for many women was noted to be an effective method for weight control [18].

Some studies found that smokers with a higher FTCD score were less likely to succeed in quitting smoking [10,19]. FTCD score was found to predict the severity of withdrawal symptoms and the need for pharmacological treatment [20]. Several factors that were related to nicotine dependence were associated with success in smoking cessation. Subjects who smoked fewer cigarettes per day and had lower concentrations of the biochemical markers of tobacco use (exhaled CO) had higher success rates [21]. CO monitors can provide biomedical feedback to assess smoking behavior, educate smokers about tobacco health effects, assist with treatment planning, and serve as a motivational tool for people to become tobacco free [22]. Our study also showed that smokers with lower level FTCD, lower exhaled CO concentration, or who on average smoked less than 20 cigarettes per day had higher success rates.

According to HPA data in 2015, the six-month cessation success rate were 26.5% and 32.3% among all healthcare institutions and medical centers, respectively [1]. In our study, we used the same criteria for smoking success and the six-month success rate was 37.7%. However, 308 out of 877 (35%) people in our study had only one visit and the average number of visit was 2.3. We compared the success rate of smokers with only one visit and those with two or more visits. We found that smokers who had two or more clinic visits had higher cessation success rates (OR = 1.37 95% CI = 1.02–1.83). Indeed, this result may imply that better compliance and motivation in the smoker is associated with a higher success rate in smoking cessation. There was also a study in Taiwan that showed total number of visits was correlated with abstinence rate [9]. Therefore, encouraging smokers to return for follow-up visits is an important task for physicians.

One study indicated that physicians’ limited knowledge of, and negative beliefs about, smoking cessation treatment was one of the reasons of physicians’ lack of engagement with smoking cessation treatment [23]. In our study, we tried to explore the association between success rate and physician’s characteristics. We found that smokers seen by physicians who on average delivered more than one smoking cessation consultations per week were more likely to quit smoking successfully than smokers seen by physicians who delivered less than one consultation per week (OR = 1.80, 95% CI = 1.27–2.57). We also found that success rate was not correlated with the length of clinical practice time of the physician, which may imply that even a young doctor could have high cessation success rates from patients if he or she provides smoking cessation consultation frequently. In addition, success rate was also not correlated with the gender of the physician.

There were some limitations in our study. First, the definition of success was self-reported smoking status that was obtained from a telephone interview with no further biochemically confirmed abstinence. Second, we used a very lenient definition of cessation success by only asking the participants whether they had smoke in the past seven days. It is difficult to compare our results with other studies using CO and harder criteria for success.

## 5. Conclusions

Smokers who had a higher FTCD, higher CO concentration, or smoked more than 20 cigarettes per day at the first visit may predict lower smoking cessation rate. Physician should put more efforts for these smokers in the outpatient smoking cessation service. Encouraging follow-up visits for smokers may also lead to higher cessation rate. Furthermore, physicians possessing more experiences in smoking cessation service seemed more likely to help patients to quit smoking successfully.

## Figures and Tables

**Table 1:** Characteristics of patients and physicians correlated with six-month smoking cessation success rate.

		6-Month Smoking Cessation Success	
Characteristics	Number (%) or Mean ± SD	Number (%) or Mean ± SD	*p* * Value
Patients	877 (100)	331 (37.7)	
Gender			0.076
Male	682 (77.8)	268 (39.3)	
Female	195 (22.2)	63 (32.3)	
Age (y)	45.3 ± 12.8	46.5 ± 13.0	0.031
Smoke-year (y)			0.942
<20	473 (53.9)	178 (37.6)	
≥20	404 (46.1)	153 (37.9)	
Cigarettes per day			0.001
<20	215 (24.5)	101 (47.0)	
≥20	662 (75.5)	230 (34.7)	
FTCD			<0.001
Low: 0–3	75 (8.6)	41 (54.7)	
Medium: 4–6	419 (47.8)	182 (43.4)	
High: ≥7	383 (43.7)	108 (28.2)	
CO	14.8 ± 11.5	12.2 ± 9.8	<0.001
Medication			0.127
Varenicline	642 (73.2)	252 (39.3)	
NRT	235 (26.8)	79 (33.6)	
Number of visit			0.026
1	308 (35.1)	101 (32.8)	
≥2	569 (64.9)	230 (40.4)	
Physicians Clinical practice			0.638
<10 years	209 (23.8)	76 (36.4)	
≥10 years	668 (76.2)	255 (38.2)	
Average weekly consultation			0.001
<1	192 (21.9)	53 (27.6)	
≥1	685 (78.1)	278 (40.6)	
Physician gender			0.647
Male	512 (58.4)	190 (37.1)	
Female	365 (41.6)	141 (38.6)	

FTCD = Fagerström test for cigarette dependence; CO = carbon monoxide; NRT = nicotine replace therapy; * Using Chi-square and *t*-test.

**Table 2:** Factors associated with six-month smoking cessation success (*N* = 877).

	6-Month Smoking Cessation Success	
	OR (95% CI)	*p* * Value
Cigarettes per day <20 vs. ≥20	1.736 (1.266–2.379)	0.001
Low FTND vs. High FTND	3.077 (1.844–5.135)	<0.001
Medium FTND vs. High FTND	1.972 (1.467–2.652)	<0.001
CO	0.960 (0.946–0.975)	<0.001
Number of visit ≥2 vs. 1	1.367 (1.021–1.831)	0.036
Physician average weekly consultation ≥1 vs. <1	1.803 (1.267–2.565)	<0.001

* Using multiple logistic regression analysis adjusting for gender and age.

## References

[1] Health Promotion Administration (2016). Taiwan Tobacco Control Annual Report. http://tobacco.hpa.gov.tw/Upload/FTB/UpFiles/2016en.pdf.

[2] Fagerstrom K. (2012). Determinants of tobacco use and renaming the FTND to the Fagerstrom Test for Cigarette Dependence. Nicotine Tob. Res..

[3] World Health Organization (WHO) (2018). Tobacco Free Initiative (TFI) 2018.

[4] Chang F.C., Hu T.W., Lin M., Yu P.T., Chao K.Y. (2008). Effects of financing smoking cessation outpatient services in Taiwan. Tob. Control.

[5] Curry S.J., Grothaus L.C., McAfee T., Pabiniak C. (1998). Use and cost effectiveness of smoking-cessation services under four insurance plans in a health maintenance organization. N. Engl. J. Med..

[6] Kaper J., Wagena E.J., Willemsen M.C., Van Schayck C.P. (2005). Reimbursement for smoking cessation treatment may double the abstinence rate: Results of a randomized trial. Addiction.

[7] Heatherton T.F., Kozlowski L.T., Frecker R.C., Fagerstrom K.O. (1991). The Fagerstrom Test for Nicotine Dependence: A revision of the Fagerstrom Tolerance Questionnaire. Br. J. Addict..

[8] Caponnetto P., Polosa R. (2008). Common predictors of smoking cessation in clinical practice. Respir. Med..

[9] Hsueh K.C., Chen C.Y., Yang Y.H., Huang C.L. (2010). Smoking cessation program in outpatient clinics of Family Medicine Department in Taiwan: A longitudinal evaluation. Eval. Health Prof..

[10] Ho K.S., Choi B.W., Chan H.C., Ching K.W. (2016). Evaluation of biological, psychosocial, and interventional predictors for success of a smoking cessation programme in Hong Kong. Hong Kong Med. J..

[11] Kottke T.E., Battista R.N., DeFriese G.H., Brekke M.L. (1988). Attributes of successful smoking cessation interventions in medical practice. A meta-analysis of 39 controlled trials. JAMA.

[12] Chang F.C., Hu T.W., Lo S.Y., Yu P.T., Chao K.Y., Hsiao M.L. (2010). Quit smoking advice from health professionals in Taiwan: The role of funding policy and smoker socioeconomic status. Tob. Control.

[13] Li L., Borland R., Yong H.H., Fong G.T., Bansal-Travers M., Quah A.C., Sirirassamee B., Omar M., Zanna M.P., Fotuhi O. (2010). Predictors of smoking cessation among adult smokers in Malaysia and Thailand: Findings from the International Tobacco Control Southeast Asia Survey. Nicotine Tob. Res..

[14] Abdullah A.S., Driezen P., Quah A.C., Nargis N., Fong G.T. (2015). Predictors of smoking cessation behavior among Bangladeshi adults: Findings from ITC Bangladesh survey. Tob. Induc. Dis..

[15] Messer K., Trinidad D.R., Al-Delaimy W.K., Pierce J.P. (2008). Smoking cessation rates in the United States: A comparison of young adult and older smokers. Am. J. Public Health.

[16] Smith P.H., Bessette A.J., Weinberger A.H., Sheffer C.E., McKee S.A. (2016). Sex/gender differences in smoking cessation: A review. Prev. Med..

[17] Wu P.C., Hsueh K.C., Mar G.Y., Hsueh S.C., Tu M.S., McRobbie H., Hajek P. (2016). Gender differences in outcome of an attempt to stop smoking among smokers attending a smoking cessation clinic in Taiwan: 3-year follow-up study. Eval. Health Prof..

[18] Perkins K.A., Levine M.D., Marcus M.D., Shiffman S. (1997). Addressing women’s concerns about weight gain due to smoking cessation. J. Subst. Abuse Treat..

[19] Dale L.C., Glover E.D., Sachs D.P., Schroeder D.R., Offord K.P., Croghan I.T., Hurt R.D. (2001). Bupropion for smoking cessation: Predictors of successful outcome. Chest.

[20] Breslau N., Johnson E.O. (2000). Predicting smoking cessation and major depression in nicotine-dependent smokers. Am. J. Public Health.

[21] Monso E., Campbell J., Tønnesen P., Gustavsson G., Morera J. (2001). Sociodemographic predictors of success in smoking intervention. Tob. Control.

[22] Goldstein A.O., Gans S.P., Ripley-Moffitt C., Kotsen C., Bars M. (2018). Use of expired air carbon monoxide testing in clinical tobacco treatment settings. Chest.

[23] Van Eerd E.A., Risør M.B., Spigt M., Godycki-Cwirko M., Andreeva E., Francis N., Wollny A., Melbye H., Van Schayck O., Kotz D. (2017). Why do physicians lack engagement with smoking cessation treatment in their COPD patients? A multinational qualitative study. NPJ Prim. Care Respir. Med..

